# Role of cAMP in the promotion of colorectal cancer cell growth by Prostaglandin E2

**DOI:** 10.1186/1471-2407-8-380

**Published:** 2008-12-19

**Authors:** Ivonne Löffler, Michael Grün, Frank D Böhmer, Ignacio Rubio

**Affiliations:** 1 Institute of Molecular Cell Biology, Centre for Molecular Biomedicine, Friedrich-Schiller-University Jena, Drackendorfer Str.1, 07747 Jena, Germany

## Abstract

**Background:**

Prostaglandin E2 (PGE2), a product of the cyclooxygenase (COX) reaction, stimulates the growth of colonic epithelial cells. It is inferred that the abrogation of prostaglandins' growth-promoting effects as a result of COX inhibition underlies the advantageous effects of non-steroidal anti-inflammatory drugs in colorectal carcinoma (CRC). Despite this appreciation, the underlying molecular mechanisms remain obscure since cell culture studies have yielded discrepant results regarding PGE2's mitogenicity.

**Methods:**

We have employed several alternative approaches to score cell proliferation and apoptosis of 4 CRC cell lines exposed to PGE2 under various conditions. To investigate the role of cAMP in PGE2's functions, activation of the cAMP pathway was assessed at different levels (changes in cAMP levels and PKA activity) in cells subjected to specific manipulations including the use of specific inhibitors or prostanoid receptor-selective agonists/antagonists.

**Results:**

Our data document that the dose-response curve to PGE2 is 'bell-shaped', with nano molar concentrations of PGE2 being more mitogenic than micro molar doses. Remarkably, mitogenicity inversely correlates with the ability of PGE2 doses to raise cAMP levels. Consistent with a major role for cAMP, cAMP raising agents and pertussis toxin revert the mitogenic response to PGE2. Accordingly, use of prostanoid receptor-selective agonists argues for the involvement of the EP3 receptor and serum deprivation of HT29 CRC cells specifically raises the levels of Gi-coupled EP3 splice variants.

**Conclusion:**

The present data indicate that the mitogenic action of low PGE2 doses in CRC cells is mediated via Gi-proteins, most likely through the EP3 receptor subtype, and is superimposed by a second, cAMP-dependent anti-proliferative effect at higher PGE2 doses. We discuss how these findings contribute to rationalize conflictive literature data on the proliferative action of PGE2.

## Background

Colorectal carcinoma (CRC) is a leading cause of cancer-based mortality in western countries, causing some 500000 annual deaths worldwide. A novel avenue of research on CRC therapy emerged some years ago as the result of a series of population-based studies which demonstrated that the long-term intake of non steroidal anti-inflammatory drugs (NSAIDs) leads to a significantly reduced risk of developing colon cancer [1]. NSAIDs such as aspirin or indomethacin are potent and selective inhibitors of cyclooxygenase (COX), of which two isoforms, COX-1 and 2, exist. Cyclooxygenase catalyzes a key step in the biosynthesis of prostaglandins (PGs), a family of bioactive lipids that regulate as diverse biological processes as inflammation, pain, immunity, nerve and bone homeostasis among many others. Over the last few years, experimental evidence stemming mostly from animal studies has accumulated to support an important contribution of COX-2 in the development of CRC [2-5]. Since COX catalyzes the opening reaction required for the biosynthesis of all PG subtypes, one major question regards the identity of the lipid mediators that transduce the pro-carcinogenic effects of COX. While studies on the function of specific PG species in the promotion of CRC have been very limited, available evidence points to a role for the PG subtype PGE2. [6-9]. For example, PGE2 elevates tumour incidence in various murine models for CRC [10-13], and cell culture experiments have implicated PGE2 and PGE2 receptor-dependent signalling in the stimulation of colon epithelial cell growth (see below).

PGE2 exerts its biological functions via binding to four types of G-protein-coupled receptors termed EP1-4 [13,14], which couple to distinct downstream second messenger systems. EP1 is a Gq-coupled receptor that elicits Ca2+ and diacylglycerol signals while EP2 and EP4 receptors are coupled to Gs-proteins and raise cAMP levels. The EP3 receptor, finally, which manifests in up to 8 splice variants, leads predominantly to the down regulation of cAMP signalling via Gi-protein-mediated inhibition of adenylate cyclase [14-16]. Which of the multiple pathways or which combination thereof emanating from the various EP receptor subtypes is responsible for the pro-carcinogenic effects of PGE2 is far from being understood. Rodent studies have implicated EP1, EP2 and EP4 receptor in intestinal tumorigenesis [13], pointing to a complex coordination of PG effects by various receptor subtypes.

In an attempt to delineate the signal transduction processes that mediate PGE2's growth-promoting effects on colon epithelial cells, a number of laboratories have carried out cell culture experiments on a few well-characterized CRC cell lines. The outcome of those studies, however, has yielded substantial discrepancies as to the growth-promoting effects of PGE2. For instance, PGE2 has been reported to induce cell proliferation of HT-29 cells in three studies [17-19], whereas two other laboratories failed to observe a proliferative effect in the same cell line [20,21]. In fact, antiproliferative effects of PGE2 on CRC cell lines have also been reported [21,22]. It is likely that these incongruencies relate to differences in the experimental protocols employed since a number of parameters including PGE2 concentration, proliferation time frame and the inclusion/exclusion of serum, among others, differ widely in the referred studies. Similarly, there is only a limited body of partially conflictive experimental data on the regulation of apoptosis in colorectal cancer cells by PGE2 [13,22-24]. In sum there is an imbalance between the appreciation of the role of COX-derived PGs in the development of CRC and our understanding of the mechanisms underlying the growth promoting effects of PGs in colon epithelial cells *in vitro*.

We have undertaken an in-depth analysis of the cell-biological effects of PGE2 on 4 commonly employed CRC cell lines. We document that PGE2 exerts a significant proliferative effect on 3 cell lines, in a dose-dependent fashion. Unexpectedly, low PGE2 dosage in the lower nano molar range fosters CRC proliferation whilst higher PGE2 concentrations do not exhibit mitogenic potency. Of note, we monitor cell proliferation in the complete absence of serum, excluding the masking of PGE2's effect by more dominant proliferative serum constituents. We furthermore present correlative and pharmacological evidence arguing that down regulation of cAMP/PKA signalling via EP3 receptor engagement is an important step of PGE2's proliferative action, suggesting that this pathway acts in a switch-like fashion to either trigger or prohibit CRC cell proliferation driven by PGE2. We further document that expression of Gi-coupled but not Gs/Gq-coupled EP3 splice variants is selectively up regulated following serum deprivation, indicating that cAMP-reducing EP3 receptor variants are regulated by the proliferative vs. quiescent status of the CRC cell. Not least, these data illustrate that EP3 receptor down regulation represents a further means by which the presence of serum may have obscured the ability of PGE2 to induce cell proliferation in previous studies. Over all, our data illustrate that mitogenic PGE2 signalling in colon epithelial cells is multi-faceted, and that the ability to induce CRC proliferation may be determined by the ability to lower cAMP signalling via Gi-coupled EP3 receptor variants, as opposed to other EP receptor types. These findings help to rationalize conflictive literature data on the *in vitro *growth-promoting effects of PGE2.

## Methods

### Materials and Reagents

Prostaglandin E2 (PGE2) was obtained from Alexis Biochemicals and dissolved in DMSO. Lysophosphatidic acid (LPA), Isoproterenol, Pertussis toxin (PTX) and propidium iodide were purchased from Sigma-Aldrich (Taufkirchen, Germany). Butaprost, Sulprostone and 11-deoxy-PGE1 were from Cayman Chemicals (Ann Arbor, USA). EP1, 3 and 4 receptor selective antagonists were kindly provided by Merck Frost, Canada [25]. [^3^H]-Thymidine (7 Ci/mmol) was obtained from Hartmann Analytic (Braunschweig, Germany). The Annexin V-binding assay kit was from BD Bioscience (Heidelberg, Germany). Antibodies against phosphorylated PKA substrates and PARP were obtained from Cell Signalling Technology (Beverly, MA). Antibody to Vinculin was purchased from BIOZOL (Eiching, Germany).

### Cell culture

The human colon cancer cell lines Caco-2, Lovo and SW480 cells were purchased from the DSMZ (Braunschweig, Germany). These cells were maintained in RPMI medium containing 10% foetal bovine serum. Early passage HT-29 cells were provided by the Institute for Nutrition (FSU Jena, Germany) and cultured in DMEM medium containing 10% foetal bovine serum. Prior to experiments, all cells were deprived of serum for 16–18 h unless otherwise stated.

### Proliferation assays

For the assessment of [^3^H]-thymidine incorporation into cellular DNA 1 × 10^4 ^HT-29, Caco-2, Lovo and SW480 cells were seeded in 24-well culture plates. 24 h later cells were deprived of serum overnight. Cells were stimulated with agonists or treated otherwise as appropriate. In cells stimulated with PGE2 and other agonists dissolved in DMSO, the final concentration of DMSO never exceeded 0.1% v/v. Once administered, PGE2-containing medium was not exchanged for fresh medium, even for longer time points of stimulation. Control experiments, in which medium was replaced by fresh PGE2-containing medium evidenced no obvious difference in the experimental outcome, arguing against PGE2 stability as a limiting factor. 12 hr prior to quenching the samples, 0,5 μCi of [^3^H]-thymidine was added to each well. Cells were washed once with ice-cold 5% TCA and incubated for 20 minutes in 5% TCA on ice. Wells were washed 3 times with ice-cold 96% ethanol, residual cell material was solubilized with (1% SDS, 2% Na_2_CO_3_, 0,1 M NaOH) and radioactivity was measured by scintillation counting.

For automated cell counting 1 × 10^5 ^HT-29, Caco-2, Lovo and SW480 cells were seeded in 12-well culture plates and cultured for 24 hours. Cells were serum-starved over night and exposed to agonists for the indicated lengths of time. Cells were detached from the culture dishes by trypsinization and counted in a CASY 1 Cell Counter (Schärfe System GmbH, Reutlingen, Germany) using the Analyzer System Model DT routine according to the manufacturer's instructions.

### Apoptosis assays

5 × 10^4 ^HT-29 cells were seeded in 6-well plates, cultured for 24 hours and serum-deprived overnight. After agonist stimulation, both attached and detached cells were collected, pooled in a vial, and lysed in 1 ml ice-cold lysis buffer A [50 mM Hepes (pH 7,5), 150 mM NaCl, 5 mM EDTA, 1% NP-40, 1 μg/ml pepstatin A, 2 μg/ml leupeptin, 1 μg/ml aprotinin, 100 μM PMSF, 100 μg/ml pefabloc]. Lysates were cleared by centrifugation at 20,000 *g *for 15 min and protein concentration was determined with the Micro BCA protein assay kit (Pierce, Bonn, Germany). Same amounts of cell extract were resolved by polyacrylamide gel electrophoresis and PARP cleavage was assessed by Western blotting. Membranes were subsequently reprobed with Vinculin to evaluate protein loading.

For flow cytometric cell cycle distribution analysis HT-29 cells were seeded at a density of 5 × 10^5 ^cells/well in 6-well culture plates for 24 hours and deprived of serum overnight. After stimulation with the indicated concentrations of PGE2 for further 120 hours cells were trypsinized, washed once with phosphate-buffered saline (PBS) and resuspended in 100 μl PBS. Annexin V-binding was determined with the assay kit following the manufacturer's instructions. Fluorescence was measured on a FACScalibur flow cytometer (Becton Dickinson, Heidelberg, Germany). The total number of cells analyzed for each sample was 10000 and raw data were processed using the CellQuestPro and WinMDI software.

### cAMP measurements

2,5 × 10^5 ^HT-29, Caco-2, Lovo and SW480 cells were seeded in 12-well culture plates and grown for 24 hours. Cells were starved of serum overnight and treated with 500 μM 3-isobutyl-1-methylxanthine (IBMX) for 4 hr followed by stimulation with the indicated concentrations of PGE2 for 15 minutes. Reactions were stopped by addition of ice cold 65% ethanol. Cells were scraped off, cell debris was pelleted by centrifugation (20,000 g, 10 min) and the supernatant was evaporated in a SpeedVac. Intracellular cAMP, present in the residue, was subsequently determined using the Cyclic AMP [^3^H] assay (Amersham Biosciences, Freiburg, Germany) exactly as described by the manufacturer.

### Phosphorylation of PKA substrates

5 × 10^6 ^cells were seeded in 6-well culture plates and cultured for 24 hours. After serum deprivation overnight cells were challenged with PGE2 for 15 minutes and lysed in 1 ml cold lysis buffer A supplemented with phosphatase inhibitors: 3,4 μM microcystin, 10 mM β-glycerophosphate, 100 μM Na-orthovanadate. Lysates were cleared by centrifugation and resolved by polyacrylamide gel electrophoresis. Phosphorylation of PKA substrates was determined by Western blotting with an anti phospho-PKA substrate antibody. The blots were subsequently reprobed with Vinculin to ascertain equal protein loading.

### RT-PCR analysis of EP receptors and EP3 receptor splice variants

For EP1-4 receptor analysis total RNA was isolated using the Rneasy Kit (Qiagen, Germany) and 1 μg was reverse-transcribed using TaqMan Reverse Transcription Reagents (Roche Diagnostics, Germany) following the manufacturers' instructions. EP receptor cDNA was amplified by standard PCR techniques using previously reported primer sets for EP1, 2, 3 (subtype 1–8) and 4 [26,27]. Amplification of EP3 receptor splice variants, along with GAPDH as an internal control in each reaction, was carried out with the OneStep reverse transcription-PCR kit from Qiagen (Hilden, Germany) according to the standard protocol with newly created primer sets for each subtype. All used primer sets are listed in table 1. A HA-tagged version of human EP3 subtype 3 (cDNA was obtained from the Missouri S&T cDNA Resource Centre) was transfected by standard procedures into HT-29 cells and used as a positive control for the PCR amplification.

**Table 1:** List of primer pairs used in the current study for amplification of EP receptor isoforms.

**EP receptor**	**Acc. No.**	**sense**	**antisense**	**fragment size in bp**	**Ref**
**EP1**	NM_000955	CTTGTCGGTATCATGGTGGTGTC	GGTTGTGCTTAGAAGTGGCTGAGG	322	[27]
**EP2**	NM_000956	CCACCTCATTCTCCTGGCTA	CGACAACAGAGGACTGAACG	216	[26]
**EP3 subtype 1–8**	NM_000957	CTTCGCATAACTGGGGCAAC	TCTCCGTGTGTGTCTTGCAG	300	[27]
**EP3 subtype 1–3**	NM_000957	CTTAATAGCTGTTCGCCTGG	GCTTAGCTGGACACTGCAG	293 (1) 224 (2) 197 (3)	This study
**EP3 subtype 4**	NM_198716	CTTAATAGCTGTTCGCCTGG	ATTTCCCCAAAATTCCTCTTG	232	This study
**EP3 subtype 5**	NM_198715	CTTAATAGCTGTTCGCCTGG	TGCTTCTGTCTGTATTATTTCAT	182	This study
**EP3 subtype 6**	NM_198716	CTTAATAGCTGTTCGCCTGG	ATTTCCCCAAAATTCCTCTTG	140	This study
**EP3 subtype 7**	NM_198717	CTTAATAGCTGTTCGCCTGG	ATTTCCCCAAAATTCCTCCTG	113	This study
**EP3 subtype 8**	NM_198718	CTTAATAGCTGTTCGCCTGG	GTCTTTACTGTTGAGATTCTG	268	This study
**EP4**	NM_000958	TGGTATGTGGGCTGGCTG	GAGGACGGTGGCGAGAAT	329	[26]

EP3 splice variant subtypes 1–3 can only be discriminated by the different size of the amplified fragments (indicated in brackets in the corresponding field).

## Results

### PGE2 stimulates colorectal cancer cell proliferation

**Figure 1:**
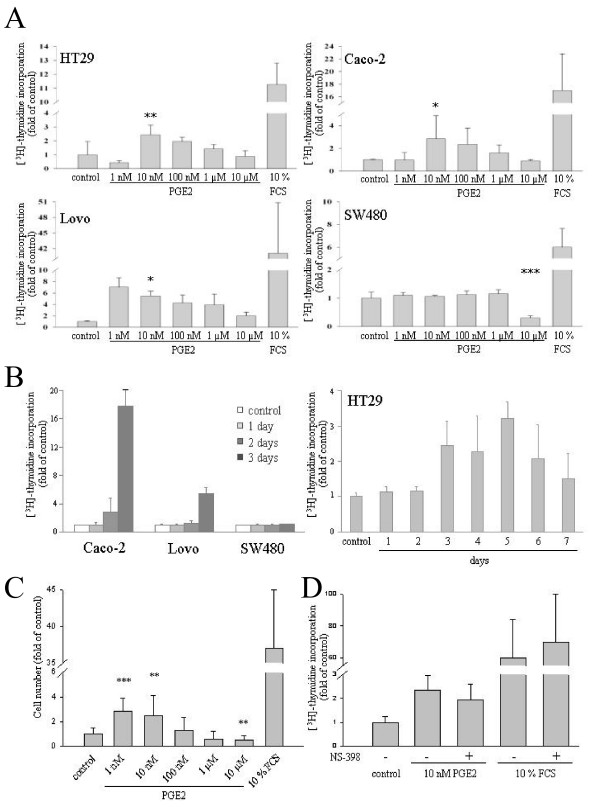
**Concentration-dependent induction of DNA synthesis by PGE2 in CRC cell lines**. A HT-29, Caco-2, Lovo and SW480 cells were seeded in 24-well plates, deprived of serum overnight and challenged with 10% FCS or the indicated doses of PGE2 for further 3 days. Proliferation was scored by [^3^H]-thymidine incorporation into cellular DNA. Data are mean ± S.E.M. of counts per minute (normalized values to average of control) of tetraplicates of three independent experiments. B Time response of PGE2-induced DNA synthesis. Serum-starved cells were exposed to 10 nM PGE2 for various days and [^3^H]-thymidine incorporation was monitored and plotted as in A. A more prolonged time response is shown for HT-29 cells in the right panel. Two further experiments yielded similar results. C PGE2-dependent cell proliferation scored by automatic cell counting. Serum-starved HT-29 cells were challenged with varying doses of PGE2 for 7 days and subjected to automatic cell counting as described in the experimental section. Data are mean ± S.E.M. of cells/well of 4 experiments with triplicate measurements. D COX-2 inhibition does not affect PGE2 induced cell proliferation. Serum-starved HT-29 cells were challenged with 10 nM PGE2 or 10% FCS alone or in combination with 10 μM NS398. 4 days later DNA synthesis was assessed as before. Two-sample comparisons (all vs. control) were performed with Student's t test. P values ***P < 0,001, **P < 0,01, *P < 0,05.

Studies on the proliferative effect of PGE2 on CRC cell lines have yielded conflictive results. This is likely due to experimental variations across the various studies in a number of parameters such as the PGE2 dose, the duration of the proliferation experiment, the presence/absence of serum, and others. Moreover, some commonly investigated cell lines manifest high heterogeneity and genetic instability [28]. To clarify the role of PGE2 on CRC cell growth, we investigated the influence of PGE2 on the proliferation of 4 CRC cell lines under various conditions. Cells were deprived of serum overnight and administered variable doses of PGE2 for 48 – 72 h. 12 h prior to quenching the samples, cell were labelled with [^3^H]-thymidine and tritium incorporation into DNA was assessed as described in the experimental section. Of note, this experiment was performed in the complete absence of serum. Under these conditions, low doses of PGE2 in the nano molar range induced significant thymidine incorporation into DNA in all cell lines except SW480 (Fig. 1A), whereas this response gradually got lost as PGE2 concentrations increased. At 10 μM or higher, PGE2 did not promote thymidine incorporation above control in any of the cell lines. In fact, in some experiments the highest dose tested (10 μM) lead to a diminished rate of DNA synthesis compared to control serum-arrested cells, in agreement with previous studies that documented an antiproliferative effect of micro molar PGE2 doses in HT-29 and other CRC cell lines [17,21,22]. Altogether, these data pointed to a concentration-dependent induction of proliferation and/or the removal of an anti-proliferative block by PGE2.

Next we monitored the time response of thymidine incorporation induced by a low dose of PGE2. Cells were serum-starved and challenged with 10 nM PGE2 for various periods of time. [^3^H]-Thymidine was administered 12 h prior to quenching the reactions. Induction of DNA synthesis by PGE2 was apparent at 72 h or later time points (Fig. 1B). PGE2 did not appreciably stimulate DNA synthesis in any of the cell types at 48 h. Thus, in contrast to the effect of serum, DNA synthesis in response to low PGE2 doses was preceded by a substantial time lag.

To confirm these findings with an alternative approach, we monitored cell proliferation by automated cell counting. Cells were serum-starved followed by addition of low (10 nM) or high (10 μM) PGE2 doses. 168 h later cell numbers were determined with an electronic cell counter (Fig. 1C). The results obtained by cell counting corroborated the data from the [^3^H]-Thymidine incorporation experiments, that is, they illustrated a proliferative effect only for low nano molar PGE2 doses.

CRC cell lines express COX-2 to varying degrees [24,29,30]. Since endogenous production of PGE2 might significantly affect the outcome of the proliferation assays, in particular in those points treated with low concentrations of exogenous PGE2, we used a COX inhibitor to negate any possible contribution of auto/paracrine effects. As illustrated in Fig. 1D, inclusion of the COX-2 inhibitor NS398 did not affect DNA synthesis induced by 10 nM PGE2 or serum. This finding indicated that endogenous prostaglandin production is not involved in the proliferative response to exogenous PGE2, a finding that is in line with literature data pointing to the absence of functional COX-2 in HT-29 cells [24,29].

### PGE2 has only minor effects on colorectal cancer cell apoptosis

**Figure 2:**
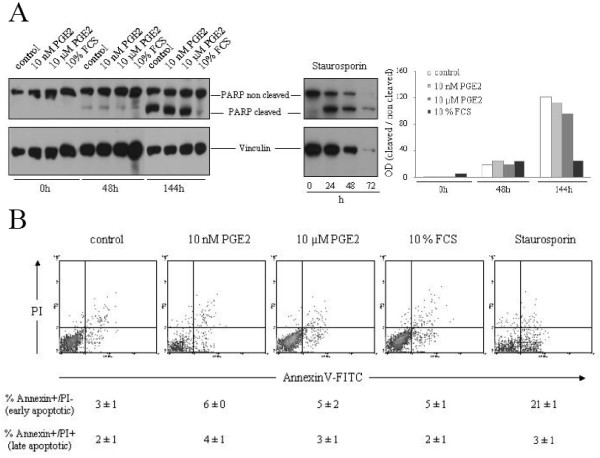
**PGE2 does not ostensibly affect apoptosis of HT-29 cells**. A HT-29 were cultured for varying lengths of time in the absence of serum or in the presence of 10% FCS, 10 nM PGE2 or 10 μM PGE2. At the indicated time points cells were lysed and PARP cleavage was assessed by western blotting. Vinculin levels were determined to ascertain equal protein loading. Staurosporine was administered at 1 μM as a positive control to induce apoptosis. Quantification is shown as the densitometrically-determined ratio of cleaved to non-cleaved PARP levels. Shown here is one representative experiment out of three. B PGE2 does not affect the number of Annexin V positive cells. HT-29 cells were treated with staurosporine, FCS or the indicated PGE2 concentrations followed by AnnexinV-FITC and propidium iodide staining and FACS analysis. The mean percentage of cells gated in the Annexin+/PI- (early apoptotic) and Annexin+/PI+ area (late apoptotic) ± S.E.M. (n = 3) is indicated below each panel.

The lack of DNA synthesis in response to micro molar PGE2 dosage could reflect anti-proliferative signalling by PGE2. Alternatively, 10 μM PGE2 might induce a higher rate of apoptosis in the CRC cell lines or reciprocally, 10 nM PGE2 could have a protective pro-survival effect under conditions of cellular stress such as serum withdrawal. To investigate this possibility we went on to measure apoptosis in HT-29 cells exposed to low (10 nM) or high (10 μM) PGE2 concentrations (Fig. 2A). Serum withdrawal for 48 h induced a weak degree of apoptosis in HT-29 cells as monitored by PARP cleavage. The broad-specificity kinase inhibitor staurosporine, which reportedly drives HT-29 cells into apoptosis [31], caused substantial PARP cleavage at this time point. Neither low or high PGE2 doses nor the presence of serum caused major alterations in the low basal rate of apoptosis in cells deprived of serum for 2 days. A marked cleavage of PARP, indicative of a robust apoptotic response, became evident only upon 144 h of serum withdrawal (Fig. 2A). This late induction of apoptosis was unaltered in cells cultured in the presence of 10 nM or 10 μM PGE2 (Fig. 2A). To confirm the lack of apoptosis regulation by PGE2, we monitored Annexin V-binding via FACS analysis (Fig. 2B). In agreement with the PARP cleavage data, serum removal induced a detectable level of apoptosis in HT-29 cells, which, however, was not affected either way by PGE2 at any dosage. We conclude that the dose-dependent differences in DNA synthesis were due to the engagement of proliferative and/or anti-proliferative rather than cell-survival pathways by PGE2.

### PGE2 affects cAMP levels in a dose-dependent way

**Figure 3:**
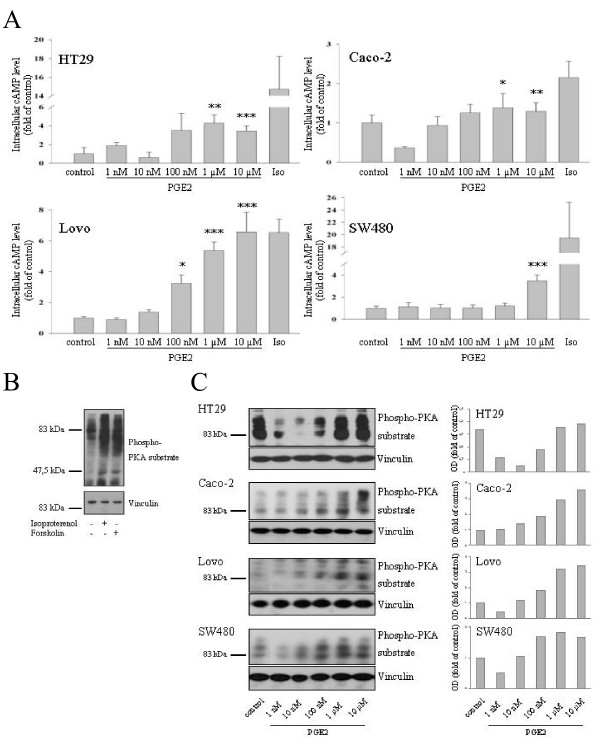
**Concentration-dependent effect of PGE2 on cAMP levels in CRC cell lines**. A Serum-starved cells were exposed to 100 μM Isoproterenol or the indicated concentrations of PGE2 for 15 min. Cyclic AMP levels were determined as described in the experimental section. B Cyclic AMP-raising agents induce phosphorylation of PKA-substrates in CRC cells. Cells were challenged with 100 μM Isoproterenol or 10 μM forskolin for 20 min and phosphorylation of PKA substrates was determined by western blotting with an antibody directed against the phosphorylated PKA-consensus site. Vinculin levels were determined to ascertain equal loading. C CRC cells were deprived of serum overnight and challenged with varying doses of PGE2 for 15 min. Phosphorylation of PKA substrates was assessed as in B. A densitometric quantification of the signal for the 70–100 kD region is shown. Two additional experiments produced essentially the same results. Two-sample comparisons (all vs. control) were performed with Student's t test. P values ***P < 0,001, **P < 0,01, *P < 0,05.

Cyclic AMP is a major intracellular mediator of PGE2 effects in numerous tissues. In colorectal cancer cell lines, cAMP has been invoked as the predominant mitogenic signalling pathway addressed by PGE2 by some laboratories [23,32,33], whereas others did not detect PGE2-dependent changes in cAMP levels of CRC cells [20]. To address the role played by cAMP in PGE2-driven cell proliferation, we measured cAMP levels in cells exposed to the various PGE2 doses tested previously for their proliferative potency in Fig. 1A. Again, the effects of PGE2 were strongly dependent on the agonist dose (Fig. 3A). Whilst low nM doses of PGE2 elicited a reduction or no changes in cAMP levels depending on the cell type, higher PGE2 dosage stimulated cAMP formation. Isoproterenol, a β-adrenergic receptor agonist and known stimulator of adenylate cyclase was used as a positive control in these experiments.

To confirm these data with an alternative approach, we assessed the phosphorylation of Protein kinase A (PKA) target proteins employing an antibody that decorates the phosphorylated PKA-consensus site on PKA substrates. PKA is a major effector protein of cAMP and, hence, monitoring PKA-dependent phosphotransfer reactions serves as surrogate readout for changes in cAMP levels. To assess the validity of this approach we first stimulated cells with isoproterenol and forskolin, two well-established cAMP raising agents. As shown in Fig. 3B, both drugs induced a similar pattern of PKA-substrate phosphorylation. Since the most prominent changes in phosphorylation occurred in the region of 60–100 kD, we henceforth focused on this region for the assessment of PGE2 effects. Serum-starved HT-29, Caco-2, Lovo and SW480 cells were exposed to various doses of PGE2 for 15 min and cell extracts were analysed by Western blotting with the anti-phospho-PKA substrate antibody. As shown in Fig. 3C, the results obtained with this approach were qualitatively similar to the data obtained by measuring cAMP levels. Low nano molar doses of PGE2 caused either no change or a reduction in PKA substrate phosphorylation, while μM PGE2 concentrations lead to a stimulation of the cAMP/PKA pathway in all 4 cell lines. Despite this major overlap, some discrepancies were observed between both assays for low PGE2 doses. Thus, in HT-29, Lovo and SW480 cells low nano molar concentrations of PGE2 reduced PKA substrate phosphorylation, in the absence of any ostensible reduction of cAMP levels. This difference probably reflects limitations of the cAMP RIA assay for the detection of small changes in cAMP levels. Alternatively, as documented recently for PGE1 [34], subcelularly restricted fluctuations in cAMP levels that may escape detection in the RIA assay, might suffice to induce activity changes in PKA and other cAMP effector pathways. Irrespective of these considerations, we conclude that PGE2 can either stimulate or down regulate cAMP signalling in colon carcinoma cells in dependency of the PGE2 dose in a manner that is counterintuitive for a role of elevated cAMP levels as a mediator of PGE2's proliferative effects.

### A rise in cAMP compromises while cAMP reducing agents promote DNA synthesis in HT-29 cells

**Figure 4:**
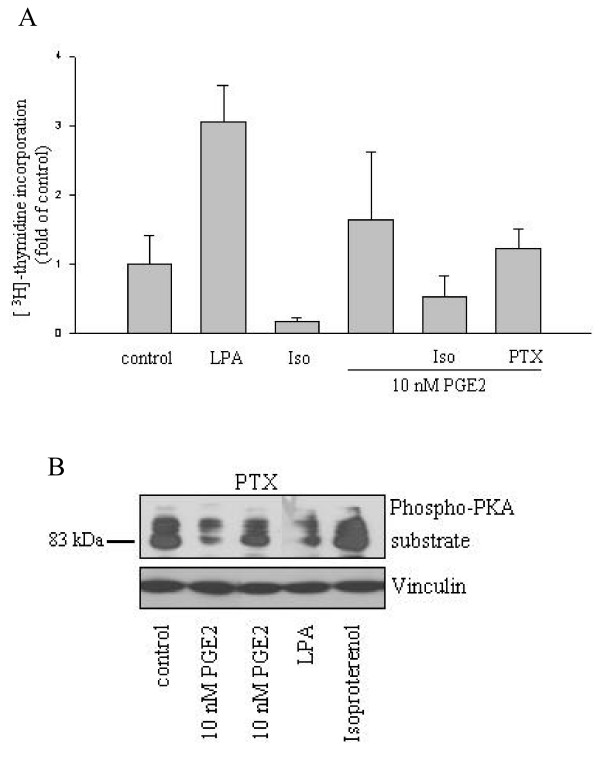
**Heterotrimeric Gi-protein-dependent signalling drives proliferation of CRC cell lines**. A 10 μM lysophosphatidic acid (LPA), 100 μM Isoproterenol, 100 ng/ml PTX and 10 nM PGE2 were administered alone or in combination to serum-starved HT-29 cells. 96 h later [^3^H]-thymidine incorporation into cellular DNA was scored as described before. Data are mean ± S.E.M. of counts per minute (normalized values to average of control) of tetraplicates. B Effect of PTX and LPA on PKA substrate phosphorylation. Serum-deprived HT-29 cells were treated as indicated in the legend and PKA-substrate phosphorylation was assessed as before.

The results presented so far highlight an inverse correlation between the ability of a particular PGE2 dose to induce cAMP signalling and its mitogenic potency on three colon carcinoma cell lines. To investigate whether cAMP signalling is a major determinant of CRC cell proliferation, we used several approaches to manipulate cAMP levels. Lysophosphatidic acid (LPA), a cAMP reducing agonist, induces the proliferation of epithelial cells types via Gi-protein-dependent signalling [35]. LPA administration caused a marked increase in DNA synthesis in HT-29 cells, consistent with a role for reduced cAMP-dependent signalling in the promotion of HT-29 cell proliferation (Fig. 4A). On the other hand isoproterenol, a cAMP raising agonist, diminished the rate of thymidine incorporation below the basal rate of serum-deprived cells. We next evaluated whether alterations in cAMP signalling were involved in the proliferative action of low PGE2 doses. As shown in Fig. 4A, induction of DNA synthesis in response to 10 nM PGE2 in HT-29 cells was fully reverted by the administration of the cAMP raising agonist isoproterenol or pertussis toxin (PTX), a specific inhibitor of heterotrimeric Gi-proteins. We ascertained that both PTX and LPA exerted the predicted effects on cAMP signalling (Fig. 4B). These data strongly indicated that down regulation of cAMP signalling was an essential component of the proliferative program evoked by PGE2. In particular, the clear effect of the highly specific reagent PTX strongly argued for the involvement of the Gi-coupled prostanoid receptor EP3.

### Pharmacological manipulation of EP receptor isoforms confirms a role for EP3/cAMP signalling in PGE2-dependent HT-29 cell proliferation

**Figure 5:**
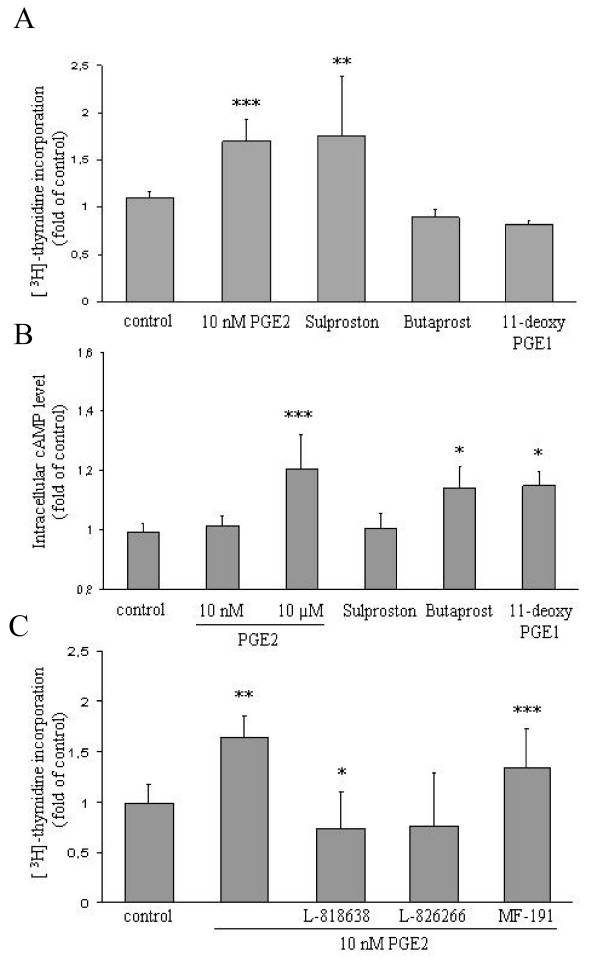
**Effect of EP3 receptor selective agonists and antagonists on HT-29 cell proliferation**. A HT-29 cells were deprived of serum overnight and challenged with 10 nM PGE2, 10 μM butaprost, 10 μM sulprostone or 10 μM 11-deoxy-PGE1. DNA synthesis was assessed 5 days later. Data are mean ± S.E.M. of cells/well of 3 experiments with triplicate measurements.B Serum-starved HT-29 cells were stimulated for 20 min with the same agonists as in A and cAMP levels were determined as before. C Serum-starved HT-29 cells were challenged with 10 nM PGE2 alone or in the presence of 250 nM each of L-818638 (EP1 antagonist), L-826266 (EP3 antagonist) or MF-191 (EP4 antagonist). Proliferation was measured 4 days later by [^3^H]-thymidine incorporation. Two-sample comparisons (all vs. control) were performed with Student's t test. P values ***P < 0,001, **P < 0,01, *P < 0,05.

To substantiate the idea that EP3 signalling mediated most if not all of PGE2's proliferative effect, we investigated the action of three prostanoid receptor selective agonists on the growth of HT-29 cells. Both the EP2 selective agonist butaprost and the EP2/4 agonist 11-deoxy-PGE1 did not elevate DNA synthesis in HT-29 cells (Fig. 5A). In fact, both drugs rather diminished the cell count after 5 days, although this effect was not significant. By contrast, the EP1/3 selective agent sulprostone, used at a concentration of 10 μM, induced a similar increase in DNA synthesis as the proliferative dose of PGE2. To ascertain the selectivity of the employed agonists, we measured the changes induced in cAMP levels (Fig. 5B). As predicted, butaprost and 11-deoxy-PGE1 induced a raise in cAMP levels, consistent with the receptor selectivity pattern. Sulprostone induced no detectable change in cAMP levels, similar to the effect of 10 nM PGE2. As discussed above, the absence of a detectable down regulation of cAMP levels by both agents at the level of cAMP is likely to result from experimental limitations or features inherent to the cAMP signalling system.

To confirm the results obtained with EP receptor selective agonists, we performed complementary experiments with EP receptor selective antagonists (Fig. 5C). 10 nM PGE2 driven proliferation was severely reduced by EP1 and EP3 specific antagonists whereas an EP4 specific blocker did not ostensibly affect proliferation in these cells.

Taken together, these findings supported the idea that low doses of PGE2 act via the EP3 receptor to induce cell proliferation through a down regulation of intracellular cAMP levels. In addition, the blockade of cell proliferation by the EP1 antagonist revealed a possible contribution of EP1 signalling to PGE2 dependent HT-29 cell proliferation.

### EP1/3 agonist stimulates and EP2/4 agonists compromise proliferation of Lovo cells

**Figure 6:**
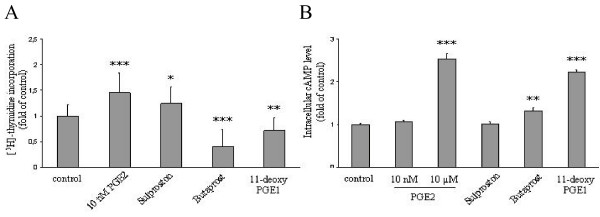
**Effect of EP3 receptor selective agonists on Lovo cell proliferation and cAMP signalling**. A HT-29 cells were deprived of serum and stimulated with 10 nM PGE2, 10 μM butaprost, 10 μM sulprostone or 10 μM 11-deoxy-PGE1. DNA synthesis was monitored 5 days later. Data are mean ± S.E.M. of cells/well of 3 experiments with triplicate measurements. B Serum-starved HT-29 cells were stimulated for 20 min with the same agonists as in A followed by measurement of cAMP levels. Student's t test: P values ***P < 0,001, **P < 0,01, *P < 0,05.

Since the experimental data presented above had centred on HT-29 as a model cell line, we wished to investigate the involvement of individual EP receptor isoforms in PGE2-dependent proliferation of a second CRC cell type. Lovo cells were serum-starved and challenged with 10 nM PGE2 or EP receptor agonists and DNA synthesis was analysed 4 days later. As shown in Fig. 6A, sulprostone and 10 nM PGE2 induced DNA synthesis in this cell line, whereas the EP2/4-agonists reduced the proliferative rate. As observed in HT-29 cells all three agonists triggered changes in cAMP that were consistent with their reported receptor selectivity profiles (Fig. 6B). These results indicate that the major role of EP3/cAMP signalling in PGE2 dependent proliferation may be a widespread phenomenon among CRC cell lines.

### Expression of the Gi-coupled prostanoid receptor subtype EP3 is growth-dependently regulated in HT-29 cells

**Figure 7:**
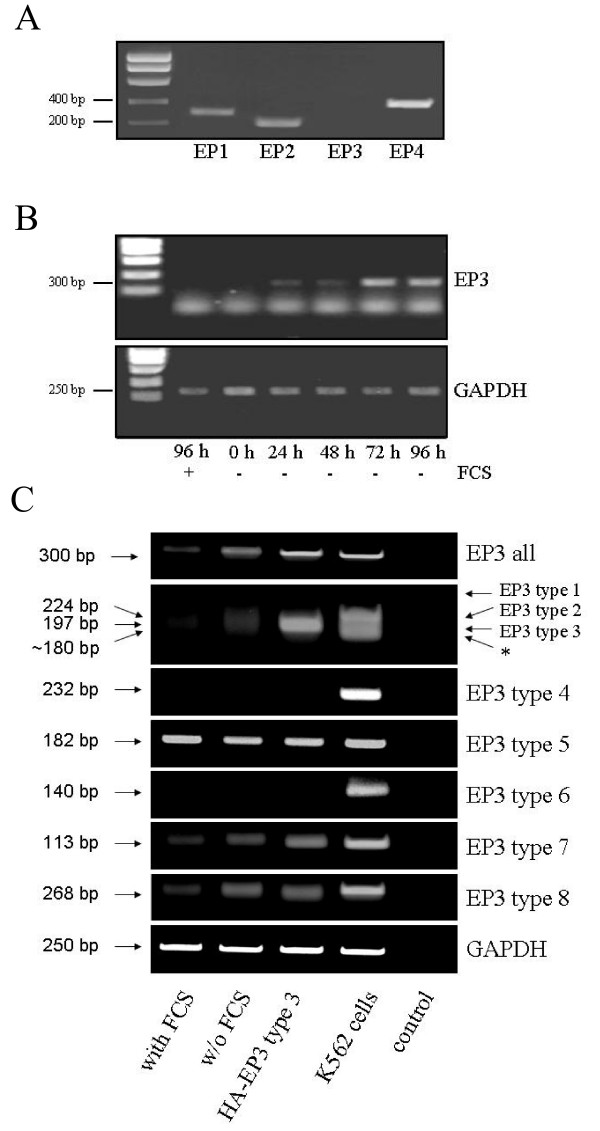
**Expression of prostanoid receptors in HT-29 cells**. A RT-PCR analysis of EP1-4 receptor expression in serum-fed HT-29 cells. See methods section and table 1 for details. Note that the primer pair employed in this experiment detects all known EP3 splice variants. B Serum-starvation induces EP3 mRNA levels. HT-29 cells were either kept in serum or serum was removed for the indicated number of days. EP3 expression was determined as in A. RT-PCR for GAPDH was run in parallel to control for equal loading. C Expression and regulation of EP3 splice variants. HT-29 cells were either kept in serum or deprived of serum for 24 h prior to performing RT-PCR on total RNA preparations. Primer pairs for individual EP3 splicing isoforms are listed in table 1. RT-PCR on total RNA from K562 cells was run as a control for EP3 isoform expression. As a further control HA-tagged EP3 subtype 3 was transfected into HT-29 cells prior to RT-PCR analysis. Note that EP3 subtypes 1–3 cannot be discriminated by use of different primer pairs but on the basis of the different size of the amplified fragments. A fragment amplified by the EP3 type 1/2/3 primer set of about 180 bp size that cannot be attributed to any known splice variant is marked by an asterisk. Control lane indicates an RT-PCR run using water as a template.

Since the preceding findings argued for a critical role of the EP3 receptor in the mediation of PGE2's proliferative effects, we wished to ascertain that HT-29 cells express this receptor subclass. To this end we performed RT-PCR analysis on total RNA preparations from HT-29 cells using primer pairs for all four EP receptor subtypes. In the case of EP3, primers were designed such as to score all *hitherto *described EP3 splice variants [16] (see experimental section and table 1). The results of this analysis, shown in Fig. 7A, evidenced that HT-29 cells express EP1, EP2 and EP4 receptors but no EP3. This result was unexpected since the effects of PTX and the EP receptor agonists/antagonists shown above were clearly indicative of the action of Gi-coupled EP3 receptors. To exclude that differences in the experimental parameters employed for the proliferation assays *versus *RT-PCR analysis could account for the lack of EP3 detection, we investigated the effect of serum withdrawal, since PGE2-dependent DNA synthesis was scored in cells deprived of serum, while RT-PCR analysis was performed on samples from serum-fed cultures. As shown in Fig. 7B serum withdrawal gradually induced the expression of EP3. Importantly, EP3 expression was detectable as early as 24 h after serum removal, that is, precisely the conditions used for cAMP signalling analysis and proliferation assays. We confirmed the identity of the EP3 PCR reaction product by sequencing (data not shown). These data supported the notion that PGE2 engages the EP3 receptor subtype to convey PTX sensitive proliferative signals via a reduction of cAMP levels in HT-29 cells.

### Gi-coupled EP3 receptor splice variants are specifically regulated by serum

The EP3 receptor manifests in 8 or possibly more splice variants, only some of which couple to cAMP reducing heterotrimeric Gi-proteins. We wished to clarify how the distinct isoforms did react to serum deprivation, since the PCR primers used in Fig. 7A/B do not discriminate among the various isoforms. Using splice variant specific primer pairs we were able to detect EP3 subtypes 3, 5, 7 and 8 and to exclude the expression of EP3 subtypes 4 and 6 in HT-29 cells. Overexpression of heterologous HA-tagged human EP3 subtype 3 prior to RT-PCR analysis was perfomed to discriminate subtypes 2 and 3. As a further control we used total RNA from K562 cells, which expressed EP3 receptor subtypes 2 and 4–8. Interestingly, K562 expressed an *hitherto *not described variant, as evidence by the 180 bp large fragment amplified by the primers for EP3 subtype 1–3. EP3 type 1 was not detectable in either HT-29 or K562 cells.

Remarkably, only the *bona fide *Gi-coupled species EP3 subtypes 7 and 8 (the downstream coupling pattern to heterotimeric G-proteins by subtype 3 is as yet unknown) [15,36-39] were induced in response to serum removal, in agreement with the current findings pointing to a major role for these receptors in PGE2 dependent cell proliferation as monitored here in the absence of serum.

## Discussion

While the beneficial action of NSAIDs in preventing colorectal cancer progression in humans is generally accepted, the molecular mechanisms underlying the pro-carcinogenic effects of its likely targets, COX and PGs, remain obscure. The present study was conducted to provide a comprehensive view of the proliferative action of PGE2 on 4 CRC cell lines in vitro. The four cell lines differ in their grade of malignancy as well as in their mutational status [40-43] and are commonly used model cell lines for mechanistic studies. Our data evidence that PGE2 elicits DNA synthesis and net proliferation in three (HT-29, Caco-2, Lovo) out of the four cell lines. PGE2 driven cell proliferation, as measured in the absence of serum by 2 independent approaches, is low compared to the mitogenic effect of serum and proceeds only after a substantial lag of 48 h in all 3 cell types. Strikingly, the dose-response curve is bi-phasic with lower concentrations of PGE2 exerting proliferation while micro molar doses are ineffective or, in the case of SW480 cells, even anti-proliferative. A similar bell-shaped response to PGE2 has been reported by Qiao and co-workers in the adenocarcinoma cell line SW1116 [17]. Similarly the related PG subtype PGE1 induces proliferation of HT-29 cells at nano molar concentrations whereas it stalls proliferation at higher dosage [44]. Along these lines, high levels of exogenously administered prostaglandins reportedly inhibit tumour cell growth or tumorigenic parameters in cell culture studies [45,46]. Yet other researchers have documented a stimulation of colon cancer cell proliferation by low concentrations of PGE2 in the nM range [17,47,48], in agreement with our own results. All these data point to a bi-faceted action of PGE2 as a stimulus (at low concentrations) and inhibitor (at high dosage) of CRC cell growth.

Since the proliferative effects of PGE2 were obtained in the absence of serum, it was important to determine whether or not the increased cell count reflected a *bona fide *mitogenic effect of PGE2 or, alternatively, a pro-survival effect that becomes evident under situations of cellular stress, such as conceivably induced by serum withdrawal. In line with this possibility, a number of reports have illustrated anti-apoptotic effects of PGE2 in CRC lines [22,23]. Two independent apoptosis assays, however, did not provide any indication for a pro-survival effect of low PGE2 doses or a pro-apoptotic effect of higher PGE2 concentrations, indicating that the effects documented herein reflect true proliferative signalling by PGE2.

Beyond raising intriguing speculations on the mechanism of action of PGE2 on colorectal cancer cell growth, the reported concentration and time dependence of PGE2's proliferative action may help to rationalize previously reported, partially conflictive findings. Cassano et al. failed to detect any effect of PGE2 on the proliferation of HT-29 cells [20]. In their study they monitored the effect of various concentrations of PGE2 on HT-29 proliferation 24 h or 48 h after PGE2 administration. However, as documented herein, an effect of PGE2 on HT-29 proliferation becomes evident not earlier than 72 h post-stimulation. Along the same lines, Parker et al. reported an anti-proliferative effect of micro molar PGE2 doses on HT-29 [21] but the same authors observed no induction of HT-29 cell proliferation by lower PGE2 dosage, in apparent discrepancy with our observations. One possible explanation could be that Parker and co-workers monitored the effect of PGE2 on HT-29 cell proliferation in the presence of 10% serum, which according to our findings is expected to obscure the proliferative effect of PGE2. On the other hand, nano molar doses of PGE2 are mitogenic for HT-29 cells kept in 2% serum [19], indicating that the amount of serum present in the assay is a critical factor when it comes to detect proliferative effects of PGE2. One parameter worth considering at this point is the regulation of the EP3 receptor by serum, as documented in the current study. In particular, the possibility that different concentrations and/or batches of serum may affect EP3 receptor subtype levels to different extents is a factor that could have a large impact on the responsiveness of CRC cell lines to PGE2. In conclusion, our data indicate that future cell-culture studies on the growth-promoting effects of prostaglandins should evaluate longer time points of PGE2 administration and a broader range of PGE2 concentrations under serum-free or low-serum conditions.

In an attempt to decipher the signalling pathways involved in PGE2's proliferative effects we have focused on the cAMP pathway. Cyclic AMP is one major second messenger system addressed by prostaglandins in numerous tissues [49]. However the role of cAMP as a potential mediator of PGE2's promotion of cell growth has been controversially discussed. Several studies document that PGE2 engages proliferative or anti-apoptotic pathways via an increase in cAMP levels [33,50]. On the other hand, down regulation of cAMP levels has been linked to PGE2-driven cell proliferation in other reports [51,52], while some laboratories have been unable to detect any PGE2-dependent changes in cAMP levels in HT-29 and other CRC cell lines, at all [20]. A recent study documents that forced signalling via the EP4 prostanoid receptor, which results in increased levels of cAMP, does not result in increased cell proliferation of HT-29 cells [53]. We find that PGE2 affects cAMP levels in all four colorectal cancer cell lines investigated here in a strictly dose-dependent fashion. Intriguingly, changes in cAMP levels in response to particular PGE2 doses inversely correlate with their proliferative potency (compare Figs. 1A and 3A): Thus, mitogenic PGE2 doses either down regulate or do not ostensibly affect cAMP levels whereas anti or non-proliferative PGE2 concentrations do in all cases elevate cAMP. Importantly, a second experimental readout for cAMP levels, the phosphorylation of substrates of the cAMP target PKA, yielded qualitatively the same results, but in several cases it evidenced a more dramatic reduction of cAMP/PKA signalling in response to mitogenic PGE2 doses than those disclosed by the RIA assay. For example, in HT-29 cells 1 nM, 10 nM and 100 nM PGE2 all lead to a reduced phosphorylation of PKA substrates, indicative of a reduction in cAMP levels, while only 10 nM PGE2 induces a detectable drop in cAMP levels as measured by the radioimmunoassay. As discussed above, several effects could account for this discrepancy. For example, PGE1 elicits cAMP accumulation at discrete sites within cells [34], suggesting that locally confined, modest changes in cAMP levels, which may be arduous to detect via RIA, may suffice to mediate changes in the activity of downstream effectors such as PKA. Moreover, since cAMP measurements are preceded by the administration of phosphodiesterase inhibitors in order to block cAMP degradation, the RIA assay may more accurately reflect raises in cAMP than a reduction in those levels. We suspect that a drop in cAMP levels, as reflected by a marked reduction in PKA substrate phosphorylation e.g. by 1 or 100 nM PGE2 in HT-29 cells (Fig. 3C) did largely pass undetected in the cAMP measurements. In conclusion, we hypothesize that a reduction in cAMP levels may be a general outcome to the administration of low PGE2 concentrations that relates to the proliferative action of nano molar PGE2 doses.

Gi-Proteins rank among heterotrimeric G-protein subclasses with the highest mitogenicity, although cell-type dependent variations do surely exist. This status is reflected by the oncogenic nature of various components of Gi-protein signalling pathways such as autotaxin, an extra cellular phospholipase A2 that generates lysophosphatidic acid (LPA), an agonist of Gi-protein coupled receptors [54], or transforming mutants of the Giα-subunits themselves [55]. In line with this notion, we document herein that the Gi-protein coupled receptor agonist lysophosphatidic acid is a strong mitogen in HT-29 cells. This shows that Gi-protein dependent signals elicit CRC cell proliferation, as corroborated by the ability of PTX to block PGE2 or LPA induced proliferation (Fig. 4B and data not shown). The major intracellular effect of Gi-protein signalling is a down regulation of cAMP levels via the inhibition of adenylate cyclase, suggesting that inhibition of cAMP signalling is an important component of the proliferative signal elicited by low PGE2 doses. Indeed, several findings reported here argue for a role of reduced cAMP dependent signalling in this context: Firstly, the Gi-protein activator LPA induces, whilst the Gi-protein inhibitor PTX inhibits CRC cell proliferation. Secondly, mitogenic doses of PGE2 down regulate cAMP/PKA signalling. Thirdly, the cAMP-raising agent isoproterenol abolishes proliferation induced by LPA and 10 nM PGE2, and finally, the pharmacological profile of PGE2 dependent mitogenesis, based on the use of receptor specific agonists and antagonists, strongly points to a major role for the down regulation of cAMP levels via Gi-proteins in PGE2 driven proliferation.

While these data all point to a role of cAMP, it is important to note that Gi-proteins can activate mitogenic pathways independently of their effect on cAMP levels. For example, LPA and other agonists of Gi-protein coupled receptors activate the Ras/Erk pathway in fibroblasts and epithelial cells independently of their effect on cAMP [56]. Several groups have documented an activation of Ras and/or its downstream target Erk by PGE2 in CRC cells [18,57,58], although the extent of those effects was weak if compared to the consequences of Gi-protein-driven Ras/Erk activation in fibroblasts or epithelial cell lines of other origin. It has been proposed that transactivation of the EGFR mediates both Ras/Erk pathway activation and stimulation of cell growth by PGE2 in CRC cell lines [18,59,60]. We have been unable to detect a significant stimulation of Ras or Erk activity by PGE2 in the cell lines studied here, even at longer time points of stimulation up to 3 h (data not shown). As a matter of fact, the cell lines studied here and in the studies referenced above do all harbour oncogenic K-Ras or B-Raf and a high constitutive activation of Erk (data not shown). In conclusion, we propose that PGE2 induces cell proliferation in CRC cells at least partly via the modulation of cAMP levels.

The prostanoid receptor subtype EP3 reportedly couples to heterotrimeric Gi-proteins and thus represents a candidate mediator of the effect of PGE2 on CRC cell proliferation. We document that HT-29 cells do express the EP3 receptor but EP3 expression appears to be tightly regulated. Removal of serum leads to the induction of EP3 expression whereas EP3 was virtually undetectable in cells kept in serum. Strikingly, among the four EP3 receptor splice variants detected in HT-29 cells, only those linked to Gi-proteins were up regulated in response to serum withdrawal, raising the question as to why CRC cells should choose to up regulate expression of G1-coupled receptors in the absence of proliferative signals. In this regard it will be intriguing to investigate how PGE2 itself regulates the expression of the distinct EP receptor isoforms and splice variants. A distinct profile of EP receptor subtypes could also explain the behaviour of SW480 cells, the only among 4 CRC cell lines studied here that did not respond to nano molar doses of PGE2 with enhanced proliferation. Interestingly, EP3 receptor levels are down regulated in colon cancer mucosa in comparison to healthy tissue [26], indicating that EP3 expression may not be compatible with a high proliferative rate in those cells. We are intrigued by the possibility that EP3 expression may be generally linked to the proliferative state of the cell and could serve as a lever to finely adjust the proliferative rate of CRC cells. According to such a scenario, and within the context of colon cancerogenesis, PGE2 signalling via EP3 could be a priming step for CRC cell mitogenesis that becomes shut off at later time points as aberrant proliferation takes over.

## Conclusion

The present study illustrates a complex behaviour of PGE2 as regulator of CRC cell *in vitro *proliferation and rationalizes previous conflictive findings on the growth promoting effects of PGE2. The strictly concentration-dependent effects of PGE2 documented herein strongly argue for two counter-regulatory effects of low versus high PGE2 doses on the regulation of CRC cell growth. Our findings argue for a down-regulation of cAMP signalling, most likely via the EP3 prostanoid receptor, as a hallmark of PGE2-driven CRC cell proliferation and provide a framework for future *in vitro *studies on the mechanism of action of prostaglandins on CRC cells.

## Abbreviations

cAMP: cyclic AMP; COX: cyclooxygenase; CRC: colorectal carcinoma; LPA: lysophosphatidic acid; NSAIDs: non steroidal anti-inflammatory drugs; PGE2: prostaglandin E2; PKA: protein kinase A; PTX: pertussis toxin; RIA: radioimmunoassay.

## Competing interests

The authors declare that they have no competing interests.

## Authors' contributions

IL carried out all experiments. MG helped with FACS analysis. FDB participated in the design and coordination of the study. IR conceived the study, participated in its design and coordination and drafted the manuscript. All authors read and approved the final manuscript.

## Pre-publication history

The pre-publication history for this paper can be accessed here:


